# Low dose mycophenolate mofetil versus cyclophosphamide in the induction therapy of lupus nephritis in Nepalese population: a randomized control trial

**DOI:** 10.1186/s12882-018-0973-7

**Published:** 2018-07-11

**Authors:** Arun Sedhain, Rajani Hada, Rajendra K. Agrawal, Gandhi R. Bhattarai, Anil Baral

**Affiliations:** 1 Nephrology Unit, Department of Medicine, Chitwan Medical College, Bharatpur, Chitwan Nepal; 2 Department of Nephrology, National Academy of Medical Sciences, Bir Hospital, Kathmandu, Nepal; 3 OptumInsight, Product Engineering and Data Solutions, Rocky Hill, CT 06067 USA

**Keywords:** Lupus nephritis, Induction therapy, Cyclophosphamide, Mycophenolate mofetil

## Abstract

**Background:**

The management of proliferative lupus nephritis (LN) comprises timely and coordinated immunosuppressive therapy. This study aimed to evaluate and compare the effectiveness and safety profile of low dose mycophenolate mofetil (MMF) and cyclophosphamide (CYC) in induction therapy of LN in Nepalese population.

**Methods:**

We conducted a prospective, open-label, randomized trial over a period of one and half years. Forty-nine patients with class III to V lupus nephritis were enrolled, out of which 42 patients (21 in each group) could complete the study. CYC was given intravenously as a monthly pulse and MMF was administered orally in the tablet form in the maximum daily dose of 1.5 g in two divided doses.

**Results:**

The mean age of the patients was 25.43 ± 10.17 years with female to male ratio of 7.3:1. Mean baseline serum creatinine was 1.58 ± 1.38 mg/dL and eGFR was 62.38 ± 26.76 ml/min/1.73m^2^. Mean 24-h urinary protein was 4.35 ± 3.71 g per 1.73 m^2^ body surface area. At 6 months, serum creatinine (mg/dL) decreased from 1.73 to 0.96 in CYC and from 1.24 to 0.91 in the MMF group with improvement in eGFR (ml/min/1.73 m^2^) from 60.33 to 88.52 in CYC and from 64.42 to 89.09 in MMF group. Twenty-four-hour urinary protein (gm/1.73m^2^) reduced from 4.47 to 0.94 in CYC and from 4.5 to 0.62 in the MMF group. Primary end point was achieved in higher percentage of patients with MMF than CYC (28.6% vs. 19%) while equal proportion of patients (67% in each group) achieved secondary end point in both groups. Number of non-responders was higher in CYC group than in the MMF group (14.3% vs. 4.8%). There was no difference in the rate of achievement of secondary end point in both CYC and MMF groups (3.16 vs. 3.05 months). The occurrence of adverse events was higher in the CYC than in MMF group (56 vs. 15 events).

**Conclusion:**

Present study has concluded that MMF, used in relatively lower dose, is equally effective in inducing remission with reduction of proteinuria and improvement of kidney function with lesser adverse events than CYC in the induction therapy of proliferative lupus nephritis.

**Trial registration:**

Retrospectively registered to ClinicalTrials.gov PRS. NCT03200002 (Registered date: June 28, 2017).

## Background

Lupus nephritis (LN), a common presentation occurring in approximately 35 to 50% of patients with systemic lupus erythematosus (SLE) [1], is characterized by an extremely heterogeneous phenomenon [2]. Management of lupus nephritis requires a timely and coordinated use of immunosuppressive therapy, which consists of induction and maintenance phases. One of the goals of management of LN is to achieve the best possible clinical efficacy with renal remission and minimal toxic effects of the immunosuppressive agents.

Effectiveness of cyclophosphamide (CYC) over corticosteroid alone in the management of LN was established in the National Institutes of Health (NIH) trials [3, 4]. The Euro–lupus nephritis trial (ELNT) demonstrated a comparable efficacy and safety profile of low-dose CYC to the high-dose NIH regimen [5]. Since then, CYC remains a reliable and effective treatment for inducing remission in lupus nephritis [6]. However, its use is associated with significant dose-dependent short- and long-term toxicity [7]. Mycophenolate mofetil (MMF), a selective lymphocyte antiproliferative agent, has emerged as one of the first-choice regimens for inducing a remission in severe active proliferative LN [8–11]. A relatively large international multicenter trial by Aspreva lupus management study (ALMS) group established the equal efficacy with relatively identical adverse effect profiles of MMF in comparison to CYC [12]. However, the dose of MMF used in this study was relatively higher and it is not clear whether low-dose MMF or CYC is superior, because these have not been compared in a head-to-head trial. The present study was aimed at comparing the efficacy and safety of low dose MMF with CYC in Nepalese LN patients.

## Methods

### Study design and subjects

This was a prospective, open-label, randomized control trial conducted in the Department of Nephrology at National Academy of Medical Sciences (NAMS), Bir Hospital, Kathmandu, Nepal, between January 2014 to June 2015. The procedures followed were in accordance with the ethical standards of the responsible committees on human experimentation (institutional and national) and with the Declaration of Helsinki Principles 1975, as revised in 2000. The study was carried out after getting approval from the institutional review board (IRB) of NAMS and has been registered to ClinicalTrials.gov PRS. (NCT03200002) with registration date of June 28, 2017. Enrolled subjects were made aware of the investigational nature of the study and informed written consent was obtained from the adult patient or the parent of the children below 18 years of age, before enrolling the participants.

**Fig. 1:**
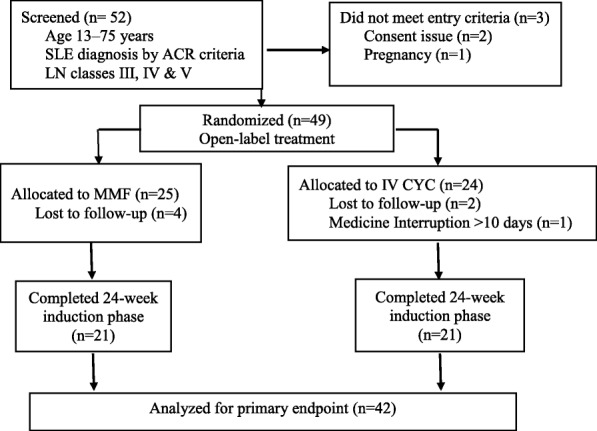
Flowchart showing patient randomization and follow-up. SLE- Systemic lupus erythematosus, LN- Lupus Nephritis, ACR- American college of rheumatology, CYC- cyclophosphamide, MMF- mycophenolate mofetil

A total of 53 patients, aged 13 years and older, diagnosed to have SLE as per American College of Rheumatology (ACR) criteria [13], and biopsy-proven class III, IV, V, III + V, or IV + V LN based on the International Society of Nephrology/Renal Pathology Society (ISN/RPS) classification [14] were screened. Patients with previous history of treatment and relapse of LN, who were receiving continuous dialysis for more than two weeks prior to randomization, who had concurrent infection or illness at the time of enrollment, female patients who were pregnant and breastfeeding and those who refused to give consent were excluded from the study. Forty-nine patients met the entry criteria and were enrolled in the study comprising of 25 and 24 patients in the MMF and CYC group respectively. The first patient was selected by a coin-toss for either MMF or CYC group and all subsequent patients were randomized alternatively in 1:1 ratio. Forty-two patients comprising 21 in each group could complete the study till the end of 6 months and were included for analysis. Those who didn’t return for follow-up or had an interruption of more than 10 days during the course of treatment were excluded from the analysis (Fig. 1).

Detailed history was taken; physical examination was done and recorded in the preformed pro-forma of the study. Investigations at baseline included complete blood count (CBC), renal function test (RFT), blood sugar level, serum calcium and phosphate, serum total protein, serum albumin, and antinuclear antibody (ANA), routine and microscopic examination of urine, 24-h urinary total protein excretion, fasting lipid profile, ultrasonography (USG) examination of the abdomen and pelvis and chest X-ray. Serum creatinine was tested by CRE2 method, which employs a modification of the kinetic Jaffe reaction with the use of SIEMENS fully automated Dimension® vista® clinical chemistry system [15]. Estimated glomerular filtration rate (eGFR) was calculated by 4-variable Modification of Diet in Renal Disease (MDRD) Study equation [16]. The clinical details including the laboratory investigation findings was recorded as baseline data.

### Study treatment and drug dosing

Patients in the MMF group were administered tablet mycophenolate mofetil at a starting dose of 750 mg twice daily if the weight was more than 50 kg. For those below 50 kg of body weight, the dose was started at 500 mg twice daily and increased to 750 mg twice daily after 30 days. The clinical response was monitored in terms of reduction in serum creatinine and 24-h proteinuria.

Those in the CYC group received the drug intravenously in the dose of 0.5 to 1 g per m^2^ of body surface area. Pulse CYC was administered every month for a total of six infusions. CYC, which is available in the powder form in a vial, was first dissolved in 20 ml of normal saline and then mixed in 100 ml of normal saline. The solution was infused over a period of one hour. At any point during the course of treatment, CYC was not given to those patients who had total leukocyte counts (TLC) less than 2500/mm^3^. Those patients were re-evaluated after one week and intravenous pulse CYC was reinstituted if the TLC increased to more than 2500/mm^3^.

Patients were monitored monthly and the clinical details were recorded. Any adverse events in between were noted and detailed physical evaluation was done and major baseline investigations except USG abdomen, chest X-ray, serum ANA and anti-double–stranded deoxyribonuclease (anti dsDNA) were repeated. If a patient had interruption of medication for more than a 10 days’ period due to any reason, the patient was withdrawn from the study.

All patients in both cohorts received concomitant corticosteroid therapy with oral prednisolone and hydroxychloroquine. Angiotensin receptor inhibitors (ACEi)/angiotensin receptor blockers (ARBs) were given to all patients if the blood pressure remained above or equal to 120 mmHg of systolic blood pressure and 80 mmHg of diastolic blood pressure. Oral prednisolone was given at an initial dose of 1 mg/kg with a maximum dose of 60 mg/day. The starting dose of prednisolone was continued for initial one month. Then, the dose of oral prednisolone was tapered at the rate of 10 mg every 2 weeks and was maintained at the baseline dose of 5 to 7.5 mg per day then after.

### Study end points

The primary outcome measure was ‘treatment response’ defined as a decrease in proteinuria- reduction of 24-h urinary total protein (UTP) to less than 3.5 g in patients with baseline nephrotic range proteinuria (UTP of ≥3.5 g) or decrease in the UTP by > 50% in patients with sub-nephrotic proteinuria (UTP < 3.5 g); or stabilization (+/− 25%) or reduction of serum creatinine and rise of eGFR from the baseline value. Secondary end point was return of serum creatinine to previous baseline, plus a decline in the 24-h UTP to less than 500 mg.

### Statistical analysis

Patient characteristics were summarized using mean and frequency distributions. Data for continuous variables were expressed as mean ± standard deviation. T-test was used to compare the mean difference of each group. Chi-square test or Fisher’s Exact test, suitable for 2 × 2 contingency table were used for test of independence. Analysis of Variation (ANOVA) tests were done to test the differences of continuous variables across multiple groups. Descriptive and inferential statistical analysis were done by using SAS University Studio package.

## Results

A total of 42 patients could complete the 6 months of study period. Details of patient recruitment is summarized in Fig. 1.

The patient age ranged from 13 to 68 years (mean 25.93 ± 10.21). There were 37 females and 5 males with female to male ratio of 7.4:1. All included baseline characteristics were comparable between two groups (Table 1). Baseline mean hemoglobin of the patients was 10.33 ± 1.95 g/dL. Mean serum creatinine was 1.47 ± 1.05 mg/dL, which was, though statistically not significant, was higher in CYC group than in MMF group (1.73 ± 1.72 vs 1.22 ± 0.53) with eGFR of 60.33 ± 28.70 ml/min and 64.42 ± 25.21 ml/min in CYC and MMF groups respectively. Although statistically insignificant, baseline disease severity indices as per SLEDAI (Systemic lupus erythematosus disease activity index) were higher in CYC group than in MMF group (Table 1).

**Table 1:** Baseline characteristics of the study population

Variables	CYC (*n* = 21)	MMF (*n* = 21)	*P*-value
Age (years)	24.67 ± 11.66	27.24 ± 9.34	0.435
Gender, n (%)			
Female	19 (90.5)	18 (85.7)	1.000
Male	2 (9.5)	3 (14.3)	
Body Mass Index (kg/m^2^)	19.64 ± 3.00	21.00 ± 3.08	0.154
BSA (m^2^)	1.48 ± 0.14	1.44 ± 1.38	0.404
SLEDAI Score	16.21 ± 4.32	15.92 ± 3.45	0.653
SLICC Criteria	11.23 ± 2.34	10.56 ± 4.78	0.562
eGFR Range (ml/min/1.73m^2^), n (%)			
≥ 90	3 (14.3)	6 (28.6)	0.381
60–89	10 (47.6)	6 (28.6)	
≤ 59	8 (38.1)	9 (42.9)	
Renal biopsy class, n (%)			
Class III/III + V	5 (23.8)	4 (19.0)	0.143
Class IV/IV + V	16 (76.2)	13 (62)	
Class V	0	4 (19.0)	
Activity index (AI)	8.09 ± 4.55	8.23 ± 3.22	2.342
Chronicity index (CI)	1.76 ± 1.72	2.61 ± 1.68	1.453
Crescents, n (%)	4 (19.04)	2 (9.52)	0.092
Serum urea (mg/dL)	62.86 ± 56.62	46.05 ± 38.05	0.266
Serum creatinine (mg/dL)	1.73 ± 1.72	1.24 ± 0.53	0.223
Positive anti-dsDNA, n (%)	17 (81.0)	19 (90.5)	0.663
Serum albumin (gm/dL)	2.75 ± 0.65	2.98 ± 0.67	0.266
Urinary 24-h protein excretion (gm)	3.32 ± 3.53	3.30 ± 2.42	0.993

*SLEDAI* Systemic Lupus Erythematosus Disease Activity Index, *SLICC* Systemic Lupus International Collaborative Clinic

According to systemic lupus international collaboration clinic (SLICC) classification criteria for SLE [17], 11 (26.2%) patients met 4 to 6 criteria, 28 (66.6%) met 7 to 9 criteria and 3 (7.14%) met ≥10 criteria. Nineteen patients in each group of CYC and MMF received angiotensin receptor blocker (ARB) and rest 2 in each group received angiotensin converting enzyme inhibitor (ACEi).

Mean activity index (AI) at baseline was 8.09 ± 4.55 in CYC and 8.23 ± 3.22 in MMF group whereas chronicity index (CI) was 1.76 ± 1.72 and 2.61 ± 1.68 in CYC and MMF groups respectively. Crescents were present in 19.04% in CYC and 9.52% in MMF group and these patients who had crescents on kidney biopsy received steroid pulse therapy at the beginning of the treatment.

## Study outcome

Achievement of primary and secondary end points in both of the treatment groups were assessed at the end of 3 and 6 months. The rates of treatment response and complete renal remission at these time points are summarized in Table 2. The average dose of the steroids used by the patients in CYC groups at the end of 3 months and 6 months was 32.74 and 12.54 mg/day and in the MMF group this dose was 31.82 and 11.46 mg/day respectively.

**Table 2:** Outcomes of treatment in two treatment groups

Efficacy measurement	End of 3 months			End of 6 months		
	CYC	MMF	*p*-value	CYC	MMF	*p*-value
Primary End point	10 (47.6%)	7 (33.3%)	0.454	4 (19.0%)	6 (28.6%)	0.572
Secondary end point	6 (28.6%)	10 (47.6%)		14 (66.7%)	14 (66.7%)	
No Response	5 (23.8%)	4 (19.0%)		3 (14.3%)	1 (4.8%)	

At the end of 3 months eGFR improved to 81.95 ± 41.28 ml/min/1.73m^2^ in CYC and 76.71 ± 28.27 ml/min/1.73 m2 in MMF group. At the end of 6 months, improvement of eGFR was 88.52 ± 35.17 and 89.09 ± 34.34 ml/min/1.73m^2^ in CYC and MMF groups respectively. Disease activity as measured by SLEDAI score also significantly improved with the treatment. SLEDAI score decreased to 6.5 ± 2.1 in CYC and 5.6 ± 2.1 in MMF group at the end of 3 months and 3.23 ± 1.32 in CYC and 2.34 ± 1.79 at the end of 6 months respectively.

At the end of 6 months, achievement of primary end point was slightly higher in MMF group than the CYC group (28.6% versus 19%), whereas achievement of secondary end point was equal in both groups (66.7% each). Three patients (14.3%) in the CYC group and 1 (4.8%) in the MMF group did not achieve response at the end of study period (6 months).

Though statistically non-significant, the average time to achieve both primary (3.21 vs. 3.5 months; *p* = 0.610) and secondary end points (3.05 versus 3.16 months; *p* = 0.817) was relatively shorter in MMF group than in the CYC group.

The treatment response rate at the end of 6 months in ISN/RPS class III and combined classes III and V was better than in classes IV and combined classes of IV and V (41.7% versus 16.7%). Secondary end point, which was achieved in 14 patients in each group, is shown in Table 3.

**Table 3:** Achievement of Secondary end point according to LN biopsy classes

Secondary end points achieved	CYC	MMF	*p*-value	Odds ratio	Confidence Interval
	No. (%)	No.%			
Baseline biopsy Class III / III + V	3 (60%)	3 (42.9%)	1.000	0.50	0.048–5.152
Baseline biopsy class IV / IV + V	11 (68.8%)	11 (78.6%)	0.689	1.67	0.318–8.741

**Fig. 2:**
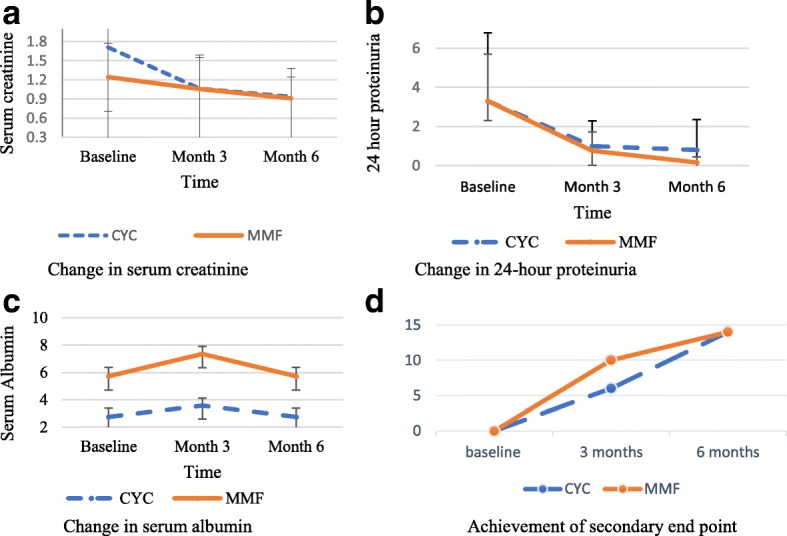
Changes in serum creatinine, serum albumin and 24-h proteinuria and achievement of secondary end point over the 6-month induction period. Serum creatinine expressed in mg/dL, 24-h proteinuria expressed in gram/day, serum albumin expressed in gram per dL and achievement of secondary end point in numbers. CYC- cyclophosphamide, MMF- Mycophenolate mofetil. **a** Change in serum creatinine. **b** Change in 24-h proteinuria. **c** Change in serum albumin. **d** Achievement of secondary end point

The changes in important renal parameters in 3 and 6 months of treatment in both groups is shown in Fig. 2.

Baseline characteristics that predicted better achievement of complete remission (secondary end point in our study) in comparison to partial remission (primary end point) were younger age of the patients (24 versus 34 years; *p* = 0.009), lesser degree of 24-h proteinuria (3.5 vs 7.45 g/1.73m^2^; *p* = 0.010) and lesser activity index on renal histology (1.68 vs. 3.7; *p* = 0.001).

### Adverse events

More than three quarters of the patients in CYC group experienced alopecia and nausea/vomiting which was absent in MMF group. Among other common adverse events, headache (38.09% vs. 19.04%) and backache (28.57% vs 19.04%) were observed by more patients in CYC group than the MMF group albeit with no statistical difference. Infection related side effects like urinary tract infection, herpes zoster and chest infection were more common in CYC group than MMF group (47.61% vs. 33.33%) (Table 4). Two patients required hospital admission for the treatment of chest infection. None of the patients died during the course of the treatment and not a single patient showed severe adverse events requiring discontinuation of treatment in both groups.

**Table 4:** Comparison of adverse events of MMF and CYC therapy

Parameter	CYC (*N* = 21)	MMF (*N* = 21)	*P* value	Odds ratio	95% Confidence interval
Alopecia	16 (76.2%)	0 (0.00%)	<.001	n/a	n/a
Nausea/Vomiting	16 (76.2%)	0 (0.0%)	<.001	n/a	n/a
Headache	8 (38.09%)	4 (19.04%)	0.778	0.423	0.084–3.456
Backache	6 (28.57%)	4 (19.04%)	0.887	0.632	0.094–4.230
Urinary tract infection	4 (19.04%)	2 (9.52%)	0.796	0.473	0.083–3.492
Herpes Zoster	3 (14.3%)	3 (14.3%)	0.337	1.000	0.178–5.632
Chest Infection	3 (14.3%)	2 (9.5%)	0.328	0.632	0.094–4.230

## Discussion

Newer therapeutic approaches to achieve best possible efficacy with lesser toxicity in the management of LN are being explored [18, 19]. Randomized, controlled trials at the National Institutes of Health (NIH) in patients with severe, proliferative LN established the better efficacy of CYC over use of corticosteroid alone but the treated subjects were not free of major adverse reactions of the drug [3]. Further studies were carried out to establish the reduced toxic profiles of CYC by using lower dose without compromising the efficacy. The Euro-lupus nephritis trial (ELNT) had compared six fortnightly injections of CYC at a fixed dose of 500 mg with high-dose monthly injections [5]. Follow-up for up to 10 years showed that there were no differences in the outcomes parameters or the side effects between high- and low-dose intravenous CYC [20].

In other studies, MMF was tested as an alternative to CYC as initial therapy of proliferative LN and it was found to be non-inferior to CYC [12, 21, 22]. Aspreva Lupus Management Study (ALMS) was a non-inferiority trial, which established almost equal results of MMF when compared with CYC in the treatment of proliferative LN [12]. In a meta-analysis of 45 trials that involved 2846 patients, there were no significant differences between CYC and MMF based induction therapy with respect to mortality, incidence of end stage renal disease (ESRD) and relapse during induction. MMF produced a numerically higher rate of complete responses (19.5 versus 13.8%), although this was not statistically significant. Major infections like pneumonia were also similar with both drugs [23]. However, the standard dose of MMF with the target dose of 3 g/d was used in all those studies. To date there have been no studies directly comparing the low-dose MMF with intravenous CYC pulse regimen.

In this randomized trial, the efficacy and safety of low dose MMF, with target daily dose of 1.5 g/d, was compared with intravenous pulse CYC in the induction phase of therapy of LN. Treatment response and complete remission rates, as well as adverse event rates were comparable in the two groups. Comparison of efficacy measurements at the end of study period between two groups has shown better achievement of primary end point in MMF than CYC groups (28.6% vs. 19%) though it was not statistically significant. This result is similar to ALMS study with achievement of primary end point in MMF and CYC (63.7% vs. 57.1%; *P* = 0.32) [12] and reported by Ginzler et al. (56.2% vs 53.0%; *P* = 0.51) [24]. However, achievement of primary end point in ALMS was much higher than in our study.

Secondary end point was achieved in equal proportion of patients in both groups (67% in each). Despite relatively worse renal function in CYC than MMF groups (serum creatinine: 1.73 mg/dL vs 1.24 mg/dL) at presentation, the achievement of secondary end point did not differ in both groups.

In our study, despite a lower received MMF dose higher response rate was achieved. The composite renal remission (combination of secondary and primary end point) was better in MMF group than CYC group (85.71% vs. 76.19%) though statistically insignificant. In ALMS report, complete remission was calculated in terms of three variables- serum creatinine, 24-h proteinuria and active urinary sediments. MMF showed better response than CYC by serum creatinine (70.3% vs 67.6%) and urinary sediments (31.4% vs. 23.8%) and lesser response by 24-h proteinuria (23.8% vs. 27%) criteria. Better response rate was seen in MMF group even on comparison of all three criteria collectively with complete remission in 8.6% in MMF and 8.1% in CYC group and partial remission 56 and 53% in these groups respectively [12]. Our study showed better composite outcome at the end of study compared to ALMS study probably due to not including urinary sediment criteria and relatively small sample size. Achievement of complete remission in our study is also better than the findings reported by Rathi et al. in their study done in Asian population, which compared MMF with low dose CYC. At the end of 24 weeks they found to have 74% of renal response in both the groups, whereas complete renal remission was achieved in 54% in the MMF group and 50% in the CYC group [25].

The composite renal response, as calculated by combination of primary and secondary end points, was slightly higher in classes III + V than classes IV + V (91.66% vs. 90.0%). Though, the explanation is not fully explainable. This result is contrary to the findings of Lupus Nephritis Collaborative Study Group, which had predicted higher likelihood of complete remission and a lower risk of renal failure in class IV LN than class III LN [26].

The occurrence of adverse events was five times more in the CYC than in MMF group (75 events vs. 15 events) in this study. Though the infection related adverse events were comparable in both groups (10 in CYC versus 7 in MMF), other milder symptoms like alopecia, nausea/vomiting and headache were more commonly observed in the CYC group (46 events vs 8 events). These findings were similar to other studies, in which MMF has been reported to be well tolerated than CYC [12, 27]. In one study pyogenic infections were found to be associated more with CYC and diarrhea more common with MMF [28]. In a recent randomized control trial, which compared MMF with low dose CYC in Indian population, although not significant, more adverse events were noted with MMF with similar infection related episodes in both groups [25]. With higher proportion of infection related to CYC use, our suggestion would be to take utmost care with close vigilance to the infection related adverse events while using CYC in the management of LN especially in this part of the world, where higher rates of various infections are prevalent.

## Strengths and limitations

This was the first study to compare lower than the conventional dose of MMF with CYC in Nepalese population. This was a single-center study. Thus, the results need validation in a large multicenter study. Our study also had a short follow-up that limits the durability of response over the long term. The study was not blinded, and this might have led to biases in recruitment of subjects or analysis of results. Larger multicenter studies with a longer follow-up are required to extrapolate these results to lupus populations in other parts of the world.

## Conclusions

Present study has concluded that low dose of MMF is as equally effective as intravenous CYC in inducing remission with reduction of proteinuria and improvement of kidney function with better safety profile in proliferative lupus nephritis in 6 months therapy in Nepalese population.

## Abbreviations

ACEi: Angiotensin receptor inhibitors
ACR: American college of rheumatology
ALMS: Aspreva lupus management study
ANA: Antinuclear antibody
ANOVA: Analysis of Variation
ARB: Angiotensin receptor blocker
CBC: Complete blood count
CYC: Cyclophosphamide
eGFR: Estimated glomerular filtration rate
ELNT: Euro lupus nephritis trial
IRB: Institutional review board
ISN/RPS: International Society of Nephrology/Renal Pathology Society
LN: Lupus nephritis
MDRD: Modification of Diet in Renal Disease
MMF: Mycophenolate mofetil
NAMS: National Academy of Medical Sciences
NIH: National Institutes of Health
RFT: Renal function test
SLE: Systemic lupus erythematosus
SLICC: Systemic lupus international collaboration clinic
USG: Ultrasonography
UTP: 24-h urinary protein
